# Commentary: An insight to PDAC tumor heterogeneity across pancreatic subregions using computed tomography images

**DOI:** 10.3389/fonc.2025.1527723

**Published:** 2025-04-28

**Authors:** Jiandong Cao, Yong Feng

**Affiliations:** Department of Thoracic Surgery, Shenyang Chest Hospital & Tenth People’s Hospital, Shenyang, Liaoning, China

**Keywords:** pancreatic ductal adenocarcinoma (PDAC), CT, pancreatic subregions, radiomics, pancreas cancer

## Introduction

I am writing to express my appreciation for the recent publication by Javed et al., titled”An insight to PDAC tumor heterogeneity across pancreatic subregions using computed tomography images” (1). This study offers a comprehensive exploration into the spatial heterogeneity of pancreatic ductal adenocarcinoma (PDAC) tumors using radiomics and machine learning techniques. The authors demonstrated that CT imaging can non-invasively differentiate tumors in the head from those in the body and tail regions, achieving promising classification accuracy. While the findings have significant implications for enhancing PDAC diagnosis and treatment strategies, I would like to offer some comments that could further enrich the discussion.

## Discussion

The authors identified distinct radiomic features that differentiate PDAC tumors based on their pancreatic subregions. However, as acknowledged in the study, the integration of imaging features with genomic and molecular profiles was not explored. Existing research has shown that combining radiomic and molecular data can lead to more precise stratification of tumors and better prediction of treatment responses (2, 3). Currently, the molecular characteristics of non-small cell lung cancer (NSCLC) form the basis for clinical diagnosis and treatment of lesions (e.g., EGFR, KRAS), and the phenotypic associations between molecular subtypes and imaging semantic features have been widely studied. A recent study on NSCLC generated 10 meaningful gene sets through RNA sequencing analysis, including the epidermal growth factor (EGF) pathway (4). An imaging-genomics map was created, with 32 statistically significant correlations between semantic imaging features and gene sets. For example, nodule attenuation and margins are associated with late cell-cycle genes, and the gene set representing the EGF pathway is significantly correlated with the presence of ground-glass opacity and irregular nodules or nodules with ill-defined margins. The authors can refer to this study to further improve their work. A deeper investigation into the correlation between radiomic features and known molecular subtypes could enhance the predictive power of the proposed model and potentially guide personalized therapeutic strategies.

The authors primarily focused on the morphological and textural characteristics of PDAC tumors without considering the tumor microenvironment (TME). The TME plays a crucial role in PDAC progression and can significantly influence imaging features (5). For instance, desmoplastic stroma, a hallmark of PDAC, might contribute to variations in signal intensity across different pancreatic regions. Non-invasive imaging techniques for characterizing the TME have not yet entered routine clinical practice. Cancer staging and treatment response assessment still mainly rely on morphological imaging, focusing on measuring the size of the primary tumor and its metastases. Positron emission tomography (PET) is a clinically established imaging technique for assessing molecular characteristics within the TME. For example, prostate-specific membrane antigen (PSMA) PET has been successfully applied in clinical practice. However, PET can only detect one molecular characteristic at a time. In contrast, magnetic resonance imaging (MRI) has the potential to more comprehensively characterize the TME, as it can be used for non-invasive evaluation of the structure, function, and molecular characteristics of tumors (6). Multiparametric MRI (mpMRI) combines multiple MRI sequences, each capturing unique features of the TME, thereby providing a more detailed insight into the tumor lesion through a single examination. Simultaneous analysis of multiple tissue characteristics more effectively addresses the complexity of the TME, including its spatial and temporal heterogeneity, and reduces the risk of missing information relevant to treatment. The authors may consider incorporating multimodal imaging techniques into their research. In the future, features related to the TME may offer a more comprehensive understanding of tumor heterogeneity.

While the Naïve Bayes classifier achieved a commendable accuracy of 86%, it is worth considering that tumor boundaries, especially between the head and body regions, may not always be well-defined. Misclassification near these boundaries suggests that anatomical overlap may influence the radiomic features. Employing advanced deep learning models or ensemble approaches might improve classification performance, particularly for tumors located at the border between subregions (7, 8). The current study analyzed CT scans from a single time point. Given PDAC’s aggressive nature, it would be valuable to investigate how radiomic features evolve over time and correlate these changes with treatment response and disease progression (9–11). Longitudinal radiomics could offer new biomarkers for early detection of treatment resistance or disease recurrence.

To translate the findings of Javed et al. into clinical practice, several practical challenges need to be addressed. First, the integration of radiomics and molecular data requires powerful computational tools as well as expertise in imaging and genomics. Collaborative efforts among radiologists, oncologists, and bioinformaticians can facilitate the development of integrated models. Second, multimodal imaging approaches, such as PET/MRI, may require additional resources and expertise. Investing in advanced imaging technologies and training healthcare professionals can help overcome these challenges. Finally, longitudinal studies require consistent follow-up and data collection. Establishing standardized protocols for data collection and analysis can ensure the reliability and reproducibility of radiomic features over time.

## Conclusion

In summary, Javed et al.’s study provides a valuable contribution to the understanding of PDAC heterogeneity using non-invasive imaging techniques. However, incorporating molecular data, TME features, and longitudinal analysis could further strengthen the study’s clinical impact. I hope the authors will consider these suggestions in their future research to expand on their promising findings.
